# Adjuvant transarterial chemoembolization following radical resection for intrahepatic cholangiocarcinoma: A multi-center retrospective study

**DOI:** 10.7150/jca.40358

**Published:** 2020-04-07

**Authors:** Lei Wang, Zi-Guo Lin, Qiao Ke, Jian-Ying Lou, Shu-Guo Zheng, Xin-Yu Bi, Jian-Ming Wang, Wei Guo, Fu-Yu Li, Jian Wang, Ya-Min Zheng, Jing-Dong Li, Shi Cheng, Wei-Ping Zhou, Yong-Yi Zeng

**Affiliations:** 1 Department of Radiation Oncology, Mengchao Hepatobiliary Hospital of Fujian Medical University, Fuzhou China, 350025; 2 Department of Hepatobiliary Surgery, Mengchao Hepatobiliary Hospital of Fujian Medical University, Fuzhou China, 350025; 3 Department of hepatobiliary surgery, the Second Hospital affiliated to Zhejiang University, Hangzhou, China, 310009; 4 Department of hepatobiliary surgery, the Southwest Hospital affiliated to the Army Medical University, Chongqing, China, 400038; 5 Department of Hepatobiliary Surgery, Cancer Hospital, Chinese Academy of Medical Sciences, Beijing, China, 100021; 6 Department of hepatobiliary surgery, Tongji Hospital affiliated to affiliated to Tongji Medical College, Huazhong University of Science &Technology, Wuhan, Hubei, China, 430030; 7 Department of Hepatobiliary Surgery, Beijing Friendship Hospital affiliated to Capital Medical University, Beijing, China, 100053; 8 Department of Hepatobiliary Surgery, the West China Hospital of Sichuan University, Chengdu, China, 610041; 9 Department of hepatobiliary surgery, Renji Hospital affiliated to Shanghai Jiaotong University, Shanghai, China, 200127; 10 Department of Hepatobiliary Surgery, Xuanwu Hospital affiliated to Capital Medical University, Beijing, China, 100050; 11 Department of Hepatobiliary Surgery, the affiliated Hospital of Chuanbei Medical University, Nanchong, China, 637000; 12 Department of Hepatobiliary Surgery, Tiantan Hospital affiliated to Capital Medical University, Beijing, China, 100050; 13 Department of Hepatobiliary Surgery Ⅲ, Eastern Hepatobiliary Surgery Hospital, Secondary Military Medical University, Shanghai China, 200438

**Keywords:** intrahepatic cholangiocarcinoma, transarterial chemoembolization, overall survival, propensity score matching

## Abstract

**Background and Aims**: The prognosis of intrahepatic cholangiocarcinoma (ICC) after radical resection is far from satisfactory, but the effect of postoperative transarterial chemoembolization (p-TACE) remains controversial. This multi-center retrospective study was to evaluate the clinical value of p-TACE and identify the selected patients who would benefit from p-TACE.

**Methods**: Data of ICC patients who underwent radical resection with/without p-TACE therapy was obtained from 12 hepatobiliary centers in China between Jan 2014 and Jan 2017. Overall survival (OS) was set as the primary endpoint, which was analyzed by the Kaplan-Meier method before and after propensity score matching (PSM). Subgroup analysis was conducted based on the established staging system and survival risk stratification.

**Results**: A total of 335 patients were enrolled in this study, including 39 patients in the p-TACE group and 296 patients in the non-TACE group. Median OS in the p-TACE group was longer than that in the non-TACE group (63.0 months vs. 18.0 months, *P*=0.041), which was confirmed after 1:1 PSM (*P*=0.009). According to the 8^th^ TNM staging system, patients with stage II and stage III stage would be benefited from p-TACE (*P*=0.021). Subgroup analysis stratified by risk factors showed that p-TACE could only benefit patients with risk factors <2 (*P*=0.027).

**Conclusion**: Patients with ICC should be recommended to receive p-TACE following radical resection, especially for those with stage II, stage III or risk factors <2. However, the conclusion deserved further validation.

## Introduction

The incidence of intrahepatic cholangiocarcinoma (ICC) is increasing stably worldwide, which accounts for 10%-15% of primary liver cancers 1, 2. The prognosis remains poor, partly because approximately half of the ICC patients have lost the chances of surgery at diagnosis 3, 4. Currently, radical resection remains the most efficient strategy for ICC 5-8, but the 5-year overall survival (OS) after radical resection is 20%-35%^9^, ^10^. Hence, postoperative adjuvant treatments are badly warranted to improve the prognosis of ICC.

Transarterial chemoembolization (TACE) has been confirmed to be efficient in the improvement of prognosis of advanced and inoperable patients 11-13, but whether postoperative TACE (p-TACE) could benefit patients following radical resection remains controversial. The clinical value of p-TACE for ICC has been evaluated in previously few studies 14-19, but it has yet reached a conclusion. However, randomized clinical trials (RCT) on this issue are rarely conducted mainly owing to the rare incidence of ICC. Hence, we collected the data from a multi-center retrospective study to evaluate the prognosis value of p-TACE for patients with ICC receiving radical resection.

## Material and Methods

### Patient selection

This study was conducted to the ethical guideline of the 1975 Declaration of Helsinki and was approved by all the participating centers including Mengchao hepatobiliary hospital, Eastern hepatobiliary surgery hospital, Affiliated Cancer Hospital of Chinese Academy of Medical Sciences, Tongji Hospital, Beijing Friendship Hospital, Xuanwu Hospital, Tiantan Hospital, the affiliated Hospital of Chuanbei Medical University, Renji Hospital, the West China Hospital, the Southwest Hospital, and the Second Hospital of Zhejiang University. Informed consent was signed by all patients or their direct relatives before surgery. Data between Jan 2014 and Jan 2017 in the 12 centers were collected via electric case report form (CRF), including baseline characteristics, operation parameters, and tumor characteristics.

### Eligibility

Patients were enrolled in this study if they 1) had a histopathologically confirmed diagnosis of ICC, 2) underwent radical resection with or without LND, 3) had no history of other malignancies, 4) had not received any preoperative anticancer therapy. Patients who had 1) incomplete clinical data, 2) preoperative obstructive jaundice, 3) extrahepatic metastasis, 4) mortality within one month after surgery, and 6) received other postoperative adjuvant therapies, such as radiotherapy, chemoradiotherapy, and immunotherapies were excluded from this study.

### Intervention

A preoperative diagnosis of ICC was primarily based on radiological findings, with or without elevated CEA and CA19-9^2^, ^6^, and liver biopsy was needed when the imaging features were not typical. The indications of surgical resection for ICC were as follows: 1) patients with performance status 0~1 before surgery; 2) tumors with or without lymph node metastasis which were evaluated to be technically resectable; 3) Child-Pugh class A to B7; 4) the estimated volume of future liver remnant was >30% in normal livers and 50% in cirrhotic livers; 5) patients without evidence of extrahepatic metastasis.

Radical resection was defined as a negative margin and without recurrence within two months after resection. All the hepatectomy and LND were conducted by highly experienced surgeons, although the procedures were a little different from each center in detail.

p-TACE was conducted only once within one to two months following resection according to the discussion of multiple discipline team. Briefly, chemotherapeutic agents including 5-fluorouracil (500 mg), epirubicin (20 mg) and hydroxycamptothecin (10 mg) were injected into the predetermined hepatic artery using a 5-F catheter, and then an emulsion of lipiodol (5-10 mL) was inserted to embolize. Of note, patients who had: 1) an Eastern Cooperative Oncology Group (ECOG) score 0-1, 2) Child-Pugh grade A or B, 3) normal kidney function, 4) white blood cell count ≥3.0 × 10^9^/L and platelet count ≥50 × 10^9^/L were eligible to receive p-TACE.

### Follow-up and definition of endpoints

All patients were periodically followed up once every 2-3 months in the first 2 years and then once every 6 months. Routine follow-up items included liver function tests, serum levels of CA19-9, CEA and AFP, and abdominal ultrasound, and a contrast-enhanced CT or MRI was warranted once recurrence was clinically suspected. Recurrence or metastasis was defined as new lesions with radiologic characteristics of ICC, and further treatment was immediately adopted whenever recurrence was confirmed. The follow-up investigation was carried out until October 2018.

The primary endpoint was overall survival (OS), and the secondary endpoint was recurrence-free survival (RFS). OS was calculated from the resection to either the date of death or the latest follow-up. RFS was defined as the time from resection to the time of recurrence (either intrahepatic or extrahepatic) or the date of the latest follow-up.

### Propensity score matching

Propensity score matching (PSM) was adopted to minify the selection bias 20, and the propensity score was determined using the potential confounding factors. Patients were then matched by a one-to-one ratio using the nearest neighbor method with a caliber of 0.2.

### Statistics

All the continuous variables were re-defined as categorical variables, hence all the variables were compared with the chi-square test or Fisher's exact test. Survival curves including OS and RFS were depicted using the Kaplan-Meier method and compared using the log-rank test. Risk factors associated with prognosis of ICC were determined by the forward method of the multivariate Cox regression model before and after PSM. Subgroup analysis was conducted based on the 8^th^ TNM staging system and risk factors.

Data analysis was conducted using SPSS 25.0, and PSM was conducted using RStudio. P<0.05 in all cases was considered statistically significant.

## Results

### Baseline characteristics

**Fig 1:**
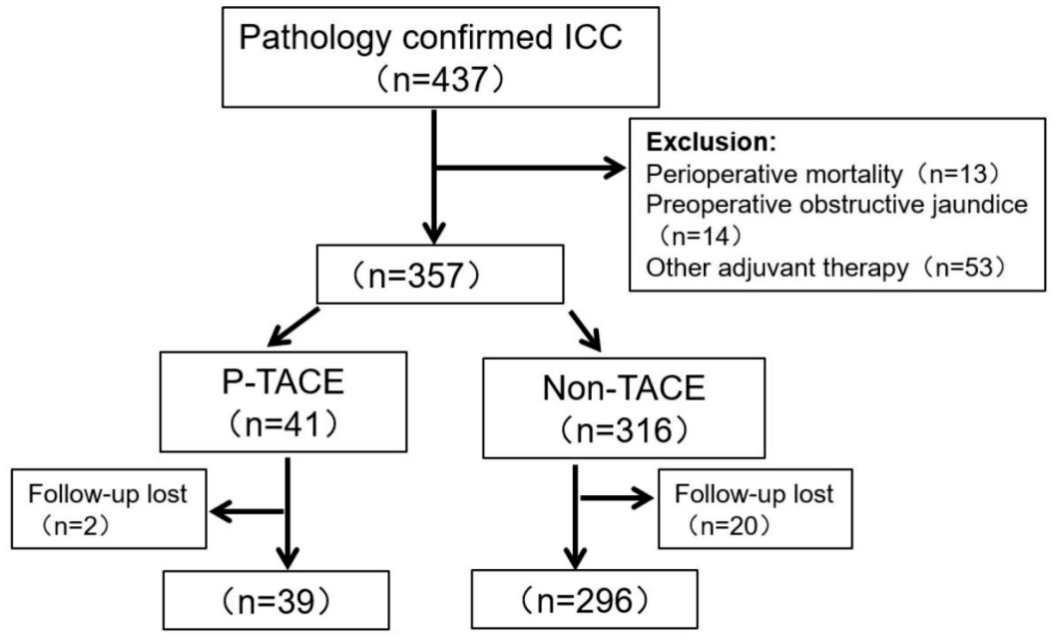
Flow chart of patients' enrollment

Initially, 437 patients with ICC underwent radical resection, but 14 patients (3.2%) were excluded for preoperative obstructive jaundice. After surgery, 13 patients (3.0%) had died within one month, and 53 (12.1%) patients received other postoperative adjuvant therapies. During the period of follow-up (1-73 months), 22 patients lost to follow-up. Finally, 335 patients were enrolled in this study, and 39 patients (11.6%) received p-TACE within two months after surgery. Detailed were depicted in Fig 1.

The baseline characteristics of the 335 patients were shown in Table 1. The median size of the resected tumor was 6.1cm, and 226 patients (67.5%) had a single tumor. 76 patients (22.7%) underwent LND, and LNM was confirmed by postoperative pathology in 41 patients (54.0%). Before PSM, patients with age <60 years, ECOG score <2, and surgical margin <1cm were more likely to receive p-TACE (all *P*<0.05, Table 1), but the clinicopathological characteristics were comparable between the two groups after 1:1 PSM (all *P*>0.05, Table 1).

### Prognosis of patients treated with or without p-TACE in the overall cohort

**Fig 2:**
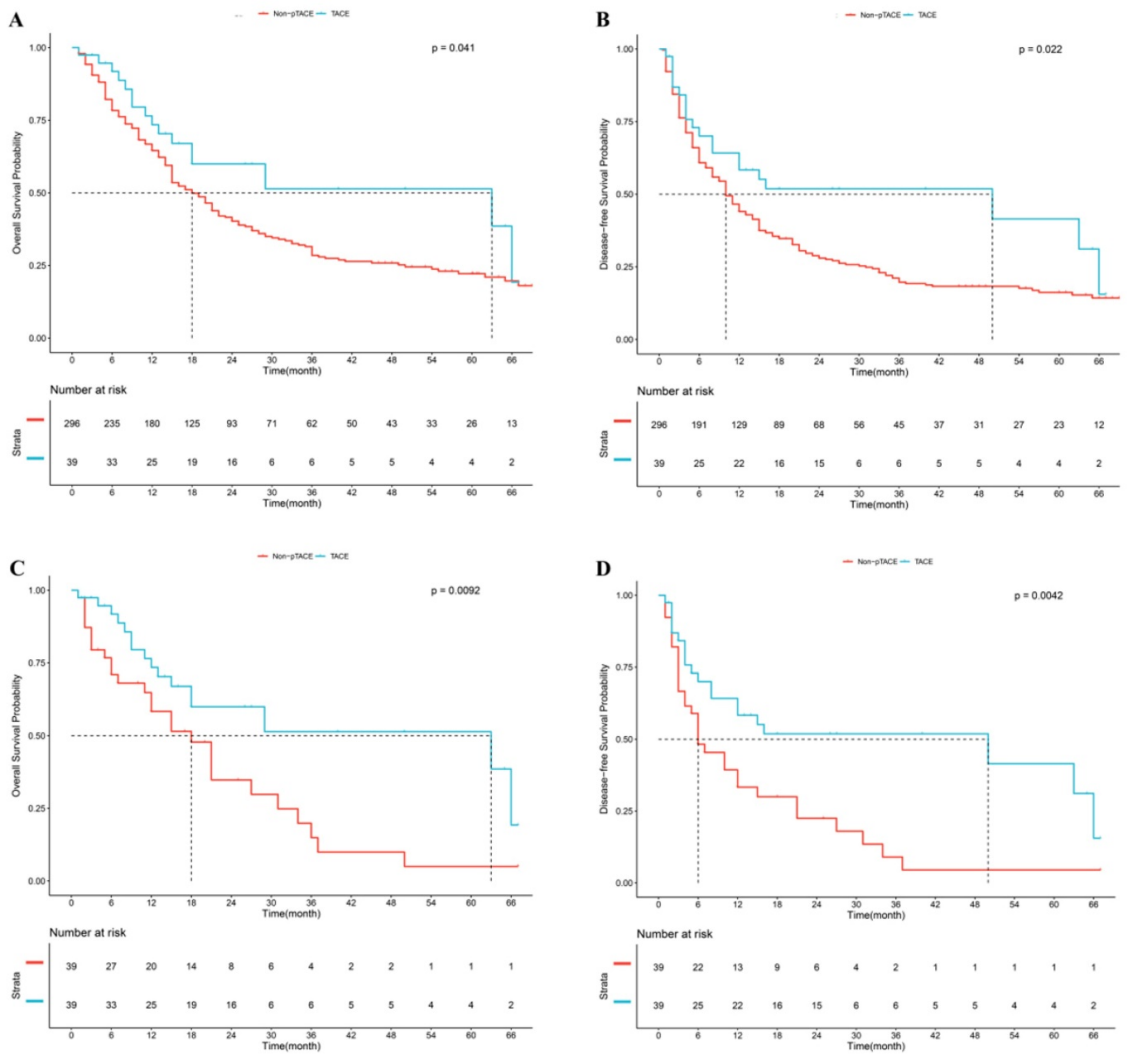
Kaplan-Meier analysis of overall survival (A) and recurrence-free survival (B) in whole cohort, Kaplan-Meier analysis of overall survival (C) and recurrence-free survival (D) after propensity score matching

In the overall study population, the mean follow-up period was 21.5 (±3.0) months in the postoperative TACE group and 21.3 (±1.1) months in the non-TACE group. Median OS in the p-TACE group was longer than that in the non-TACE group (63.0 months vs. 18.0 months, *P*=0.041, Fig 2A). The 1-, 2-, and 3-year survival rates in the p-TACE group were higher than those in the non-TACE group (76.9% vs. 65.9%, *P*=0.167; 66.7% vs. 46.0%; *P*=0.015; 64.1% vs. 37.8%; *P*=0.002; respectively). Median RFS in the p-TACE group was longer than that in the non-TACE group (50.0 months vs. 10.0 months, *P*=0.022, Fig 2B). The 1-, 2-, and 3-year RFS rates in the p-TACE group in the p-TACE group and the non-TACE group were 61.5% vs. 45.3% (*P*=0.056); 56.4% vs. 31.8% (*P*=0.002); 56.4% vs. 25.7% (*P*<0.001); respectively.

After 1:1 PSM, median OS in the p-TACE group was longer than that in the non-TACE group (63.0 months vs. 18.0 months, *P*=0.009, Fig 2C). The 1-, 2-, and 3-year survival rates in the p-TACE group were significantly higher than those in the non-TACE group (76.9% vs. 61.5%, *P*=0.141; 66.7% vs. 46.2%; *P*=0.068; 64.1% vs. 35.9%; *P*=0.013; respectively). Median RFS in the p-TACE group was longer than that in the non-TACE group (50.0 months vs. 6.0 months, *P*=0.004, Fig 2D). The 1-, 2- and 3-year RFS rates in the p-TACE group were significantly higher than those in the non-TACE group (61.5% vs. 35.9%, 56.4% vs. 28.2%, 56.4% vs. 20.5%, respectively, all *P*<0.05).

### Risk factors associated with overall survival before and after PSM

CA19-9 (HR=1.458, 95% CI=1.068~1.920, *P*=0.018), LNM (HR=1.897, 95% CI=1.116~3.237, *P*=0.018), tumor size (HR=1.621, 95% CI=1.186~2.213, *P*=0.002), and satellite (HR=1.826, 95% CI=1.103~2.976, *P*=0.019) were identified as independent risk factors for OS in a whole cohort (Table 2). After 1:1 PSM, tumor size (HR=2.121, 95% CI=1.123~4.011, *P*=0.021), satellite (HR=2.189, 95% CI=1.163~4.144, *P*=0.016) and p-TACE (HR=0.493, 95% CI=0.264~0.911, *P*=0.025) were identified as independent risk factors for OS in a whole cohort (Table 3).

### Effect of p-TACE based on the 8^th^ TNM staging system

**Fig 3:**
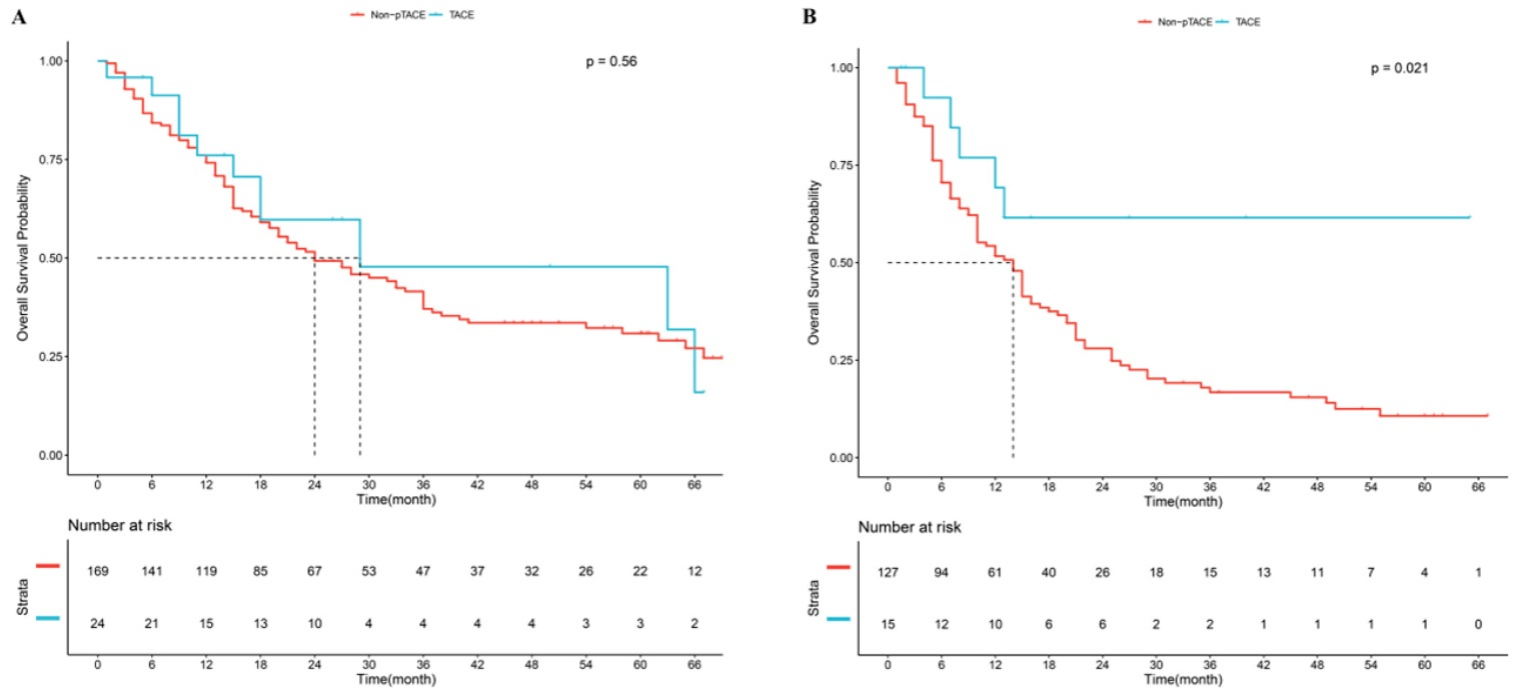
Kaplan-Meier analysis of overall survival based on 8^th^ AJCC staging system. (A), subgroup of patients with stage I, (B) subgroup of patients with stage II and stage III

In the 8^th^ TNM staging system, good prognostic stratification was observed among stage I, stage II and stage III (P<0.05, Supplement Fig 1A). Considering patients in the stage II and stage III receiving p-TACE were too small, so we took them into one subgroup. Results showed that in the subgroup of patients with stage I, no significant difference was observed between p-TACE group and non-TACE group (*P*=0.560, Fig 3A); while in the subgroup of patients with stage II and stage III, significant difference was found between p-TACE group and non-TACE group (*P*=0.021, Fig 3B).

### Effect of p-TACE based on risk factors

**Fig 4:**
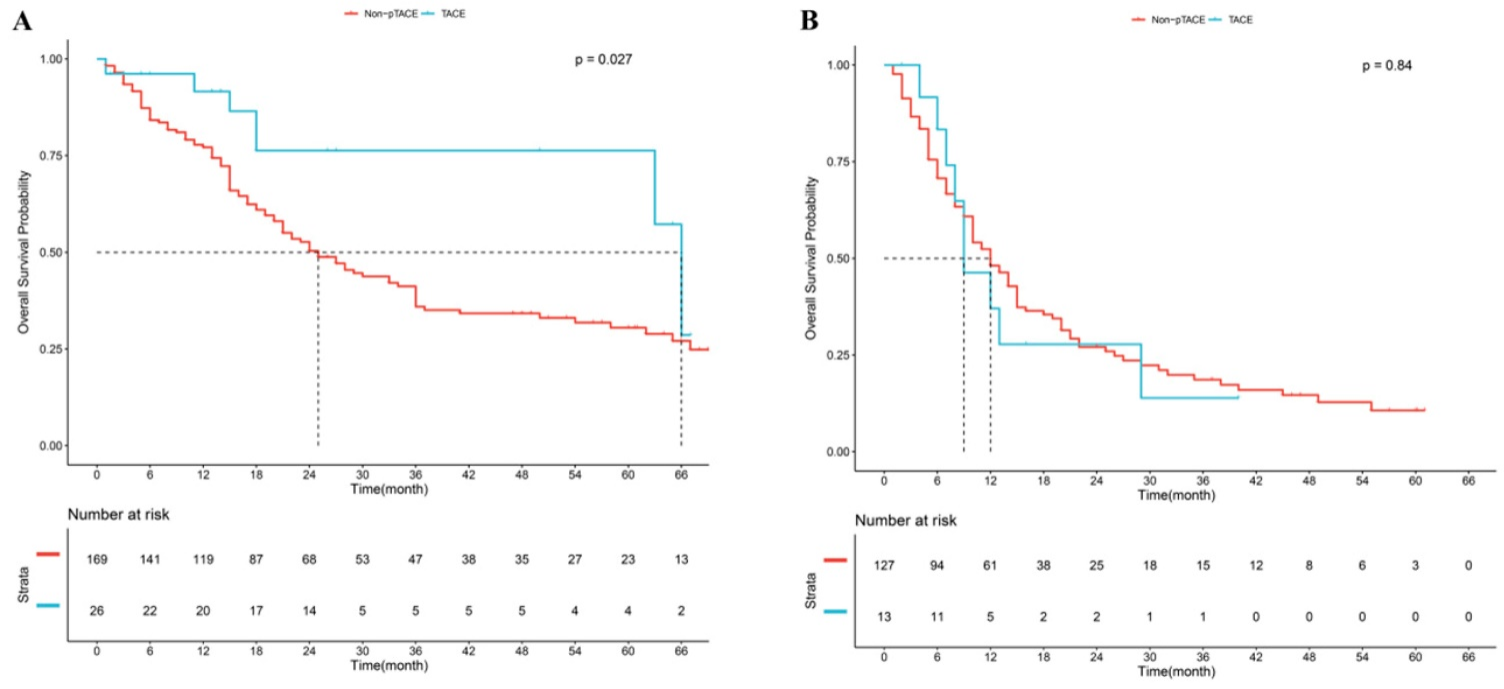
Kaplan-Meier analysis of overall survival based on risk factors. (A), subgroup of patients with “low risk”, (B) subgroup of patients with “high risk”.

CA19-9, LNM, tumor size, and satellite were confirmed to be independent risk factors of OS, and patients were divided into “high risk” and “low risk” subgroups according to the number of risk factors. Results showed that good prognostic stratification was observed between patients with risk factors <2 and patients with risk factors ≥2 (*P*<0.05, Supplement Fig 1B). Further analysis showed that in the subgroup of patients with risk factors <2, significant difference was observed between p-TACE group and non-TACE group (*P*=0.027, Fig 4A); while in the subgroup of patients with risk factors ≥2, no significant difference was found between p-TACE group and non-TACE group (*P*=0.840, Fig 4B).

## Discussion

The prognosis of ICC after radical resection remains poor 5, 7, 21, and strategies intended to reduce early recurrence and improve the long-term prognosis are still badly warranted. p-TACE has been tried with the aim of anti-recurrence, but its efficacy remains controversial^14^-^19^. In this study, 39 of 335 patients (11.6%) received p-TACE following radical resection, which was lower than that in the previous reports 16, 18. Results showed that patients in the p-TACE group enjoyed longer median OS and RFS than those in the non-TACE group before and after PSM (all P<0.05). Hence, TACE should be conducted after radical resection for ICC.

Radical resection is still the first-line treatment for patients with ICC 5-7, although half of the patients have lost the chances of resection at diagnosis 3, 4. However, the median OS of ICC after radical resection has been reported to be 21.0-39.0 months 10, 22, 23, which is far from satisfactory. Reasons might be as follows: 1) aggressive characteristics of ICC 24, 2) high incidence of LNM but low incidence of LND 25, 26, and 3) high rate of early recurrence 27, 28. In this study, the incidences of LND and LNM were 22.7%, and 54.0%, respectively, and the rate of recurrence within two years after radical resection was 61.5%. Hence, more strategies should be considered to improve the prognosis of ICC.

TACE is often considered as one of the important postoperative adjuvant therapies for primary liver cancers 29, 30, and has been conducted prevalently worldwide 31, 32. Currently, few studies reported the clinical value of p-TACE for ICC 14-19, but conclusion has yet to be reached. In this study, the benefit of p-TACE group was observed in the whole cohort (*P*<0.05), and it was confirmed after 1:1 PSM (*P*<0.05), which indicated that our results were very convincing.

However, one size was not fit for all. Previously, only patients with advanced stage or scores ≥77 based on the established ICC nomogram were reported to be benefited from p-TACE 16, 19. In this study, we found that only patients with stage II and stage III according to the 8^th^ TNM staging system would be benefited from p-TACE, which was consistent with previous reports. However, query remains, are patients with “high risk” benefited from prophylactic p-TACE? In this study, subgroup analysis showed that only patients with risk factors <2 would be benefited from p-TACE, rather than those with risk factors ≥2. In our opinion, patients with “high risk” were more likely to relapse, and need more aggressive strategies.

### Limitations

Nevertheless, there were several restrictions in this study. First, it was a retrospective study, and recalling bias was inevitable. Second, confounding factors related to the efficacy of p-TACE were almost inevitable, although a well-designed PSM was carried out. Thirdly, the incidence of patients receiving p-TACE was low (39/335, 11.6%), which was not optimal to reach a robust conclusion. The last but not the least, patients receiving p-TACE were typical present with aggressive characteristics and/or not sensitive to chemotherapy.

## Conclusion

In summary, p-TACE would benefit patients with ICC receiving radical resection, especially for those with stage II, stage III or risk factors <2. However, the conclusion requires further validation.

## Supplementary Material

Supplementary figure.

## Figures and Tables

**Table 1:** Baseline Characteristics before and after PSM

		Before PSM				After PSM		
		Non-TACE	p-TACE	*P*-Value		Non-TACE	p-TACE	*P*-Value
		(n=296)	(n=39)			(n=39)	(n=39)	
**Gender**	Female	110 (37.2%)	13 (33.3%)	0.772		10 (25.6%)	13 (33.3%)	0.619
	Male	186 (62.8%)	26 (66.7%)			29 (74.4%)	26 (66.7%)	
**Age**	<60years	176 (59.5%)	32 (82.1%)	0.011		32 (82.1%)	32 (82.1%)	1.000
	≥60years	120 (40.5%)	7 (17.9%)			7 (17.9%)	7 (17.9%)	
**Hepatitis**	No	193 (65.2%)	20 (51.3%)	0.128		25 (64.1%)	20 (51.3%)	0.359
	Yes	103 (34.8%)	19 (48.7%)			14 (35.9%)	19 (48.7%)	
**ECOG grade**	<2	236 (79.7%)	37 (94.9%)	0.039		38 (97.4%)	37 (94.9%)	1.000
	≥2	60 (20.3%)	2 (5.1%)			1 (2.6%)	2 (5.1%)	
**CA19-9**	≤37U/L	213 (72.0%)	26 (66.7%)	0.618		24 (61.5%)	26 (66.7%)	0.813
	>37U/L	83 (28.0%)	13 (33.3%)			15 (38.5%)	13 (33.3%)	
**TBil**	≤20µmol/L	155 (52.4%)	18 (46.2%)	0.576		13 (33.3%)	18 (46.2%)	0.355
	>20µmol/L	141 (47.6%)	21 (53.8%)			26 (66.7%)	21 (53.8%)	
**Child-Pugh**	A	187 (63.2%)	28 (71.8%)	0.380		30 (76.9%)	28 (71.8%)	0.795
	B	109 (36.8%)	11 (28.2%)			9 (23.1%)	11 (28.2%)	
**Blood loss**	≤400mL	244 (82.4%)	35 (89.7%)	0.357		30 (76.9%)	35 (89.7%)	0.224
	>400mL	52 (17.6%)	4 (10.3%)			9 (23.1%)	4 (10.3%)	
**Transfusion**	No	260 (87.8%)	36 (92.3%)	0.581		30 (76.9%)	36 (92.3%)	0.117
	Yes	36 (12.2%)	3 (7.7%)			9 (23.1%)	3 (7.7%)	
**Margin**	Wide	69 (23.3%)	18 (46.2%)	0.004		18 (46.2%)	18 (46.2%)	1.000
	Narrow	227 (76.7%)	21 (53.8%)			21 (53.8%)	21 (53.8%)	
**Differentiation**	Well &Moderate	247 (83.4%)	34 (87.2%)	0.716		28 (71.8%)	34 (87.2%)	0.161
	Poor	49 (16.6%)	5 (12.8%)			11 (28.2%)	5 (12.8%)	
**Tumor Number**	Single	199 (67.2%)	27 (69.2%)	0.945		28 (71.8%)	27 (69.2%)	1.000
	Multiple	97 (32.8%)	12 (30.8%)			11 (28.2%)	12 (30.8%)	
**Tumor size**	≤5cm	101 (34.1%)	21 (53.8%)	0.026		21 (53.8%)	21 (53.8%)	1.000
	>5cm	195 (65.9%)	18 (46.2%)			18 (46.2%)	18 (46.2%)	
**Satellite**	No	204 (68.9%)	29 (74.4%)	0.611		30 (76.9%)	29 (74.4%)	1.000
	Yes	92 (31.1%)	10 (25.6%)			9 (23.1%)	10 (25.6%)	
**Neurological invasion**	No	277 (93.6%)	38 (97.4%)	0.551		33 (84.6%)	38 (97.4%)	0.113
	Yes	19 (6.4%)	1 (2.6%)			6 (15.4%)	1 (2.6%)	
**LNM**	No	261 (88.2%)	33 (84.6%)	0.706		34 (87.2%)	33 (84.6%)	1.000
	Yes	35 (11.8%)	6 (15.4%)			5 (12.8%)	6 (15.4%)	
**MVI**	No	268 (90.5%)	37 (94.9%)	0.554		37 (94.9%)	37 (94.9%)	1.000
	Yes	28 (9.5%)	2 (5.1%)			2 (5.1%)	2 (5.1%)	
**AJCC**	I	169 (57.1%)	24 (61.5%)	0.547		23 (59.0%)	24 (61.5%)	0.855
	II-III	127 (42.9%)	15 (38.5%)			16 (241.0%)	9 (38.5%)	

**Abbreviations**: PSM, propensity score matching; ECOG, the Eastern Cooperative Oncology Group; TB, total bilirubin; LNM, lymph node metastasis; MVI, microvascular invasion; AJCC, American joint committee on cancer staging; p-TACE, postoperative transarterial chemoembolization.

**Table 2:** Univariate and multivariate analysis of overall survival for patients with intrahepatic cholangiocarcinoma in a whole cohort

	Univariate analysis			Multivariate analysis		
	HR	95%CI	*P*	HR	95%CI	*P*
**Gender (Female/ Male)**	1.244	0.935-1.655	0.134			
**Age (<60 years vs ≥60 years)**	1.133	0.862-1.490	0.370			
**Hepatitis (No vs Yes)**	0.874	0.659-1.159	0.349			
**ECOG grade (<2 vs ≥2)**	1.342	0.969-1.858	0.077			
**CA19-9 (≤37U/L vs >37U/L)**	1.549	1.158-2.075	0.003	1.458	1.068-1.920	0.018
**TBil (≤20µmol/L vs >20µmol/L)**	0.855	0.653-1.120	0.256			
**Child-Pugh (A vs B)**	1.049	0.786-1.375	0.755			
**Blood loss (≤400mL vs >400mL)**	1.158	0.805-1.656	0.429			
** Transfusion (No vs Yes)**	1.360	0.907-2.039	0.136			
**Margin (Wide vs Narrow)**	1.318	0.945-1.829	0.097			
**Differentiation (Well &moderate vs Poor)**	1.258	0.868-1.815	0.224			
**Tumor number (Single vs Multiple)**	1.658	1.245-2.187	<0.001			
**Tumor size (≤5cm vs >5cm)**	1.729	1.276-2.315	<0.001	1.621	1.186-2.213	0.002
**Satellite (No vs Yes)**	1.946	1.468-2.588	<0.001	1.826	1.103-2.976	0.019
**Neurological invasion (No vs Yes)**	1.231	0.715-2.120	0.453			
**LNM (No vs Yes)**	1.905	1.282-2.831	0.001	1.897	1.116-3.237	0.018
**MVI (No vs Yes)**	1.515	0.978-2.336	0.065			
**AJCC (I vs II-III)**	1.648	1.226-2.227	0.001			
**p-TACE (No vs Yes)**	0.597	0.358-0.994	0.047			

**Abbreviations**: HR, hazard ratio; CI, confidence interval; TB, total bilirubin; LNM, lymph node metastasis; MVI, microvascular invasion; AJCC, American joint committee on cancer staging; p-TACE, postoperative transarterial chemoembolization.

**Table 3:** Univariate and multivariate analysis of overall survival for patients with intrahepatic cholangiocarcinoma after propensity score matching

	Univariate analysis			Multivariate analysis		
	HR	95%CI	*P*	HR	95%CI	*P*
**Gender (female/male)**	1.642	0.786-3.387	0.179			
**Age (<60 years vs ≥60 years)**	1.392	0.638-3.021	0.411			
**Hepatitis (No vs Yes)**	1.221	0.662-2.263	0.522			
**ECOG grade (<2 vs ≥2)**	0.373	0.051-2.711	0.328			
**CA19-9 (≤37U/L vs >37U/L)**	1.958	1.051-3.567	1.842			
**TBil (≤20µmol/L vs >20µmol/L)**	0.656	0.364-1.211	0.176			
**Child-Pugh (A vs B)**	1.011	0.513-1.989	0.978			
**Blood loss (≤400mL vs >400mL)**	1.243	0.514-2.998	0.632			
**Transfusion (No vs Yes)**	1.551	0.684-3.53	0.294			
**Margin (Wide vs Narrow)**	0.911	0.489-1.656	0.752			
**Differentiation (Well &moderate vs Poor)**	2.042	0.956-4.325	0.063			
**Tumor Number (Single vs Multiple)**	1.222	0.643-2.321	0.543			
**Tumor size (≤5cm vs >5cm)**	1.986	1.068-3.737	0.031	2.121	1.123-4.011	0.021
**Satellite (No vs Yes)**	2.387	1.278-4.465	0.006	2.189	1.163-4.144	0.016
**Neurological invasion (No vs Yes)**	2.285	0.947-5.512	0.064			
**LNM (No vs Yes)**	1.312	0.514-3.368	0.578			
**MVI (No vs Yes)**	0.662	0.158-2.812	0.578			
**AJCC (I vs II-III)**	0.889	0.442-1.816	0.757			
**p-TACE (No vs Yes)**	0.438	0.241-0.834	0.011	0.493	0.264-0.911	0.025

**Abbreviations:** HR, hazard ratio; CI, confidence interval; TB, total bilirubin; LNM, lymph node metastasis; MVI, microvascular invasion; AJCC, American joint committee on cancer staging; p-TACE, postoperative transarterial chemoembolization.

## Abbreviations

ICC: intrahepatic cholangiocarcinoma
TACE: transarterial chemoembolization
PSM: propensity score matching
LND: lymph node dissection
LNM: lymph node metastasis
OS: overall survival
RFS: recurrence-free survival
HR: Hazard ratio
OR: odd ratio
CI: confidence interval
