# Supplementary material for: Adjuvant transarterial chemoembolization following radical resection for intrahepatic cholangiocarcinoma: A multi-center retrospective study
Source: J Cancer. 2020 Apr 7;11(14):4115–22. doi: 10.7150/jca.40358 (PMC7196258; doi:10.7150/jca.40358)

Supplement figure. Kaplan-Meier analysis of overall survival based on 8th AJCC staging system (A) and risk factors (B).

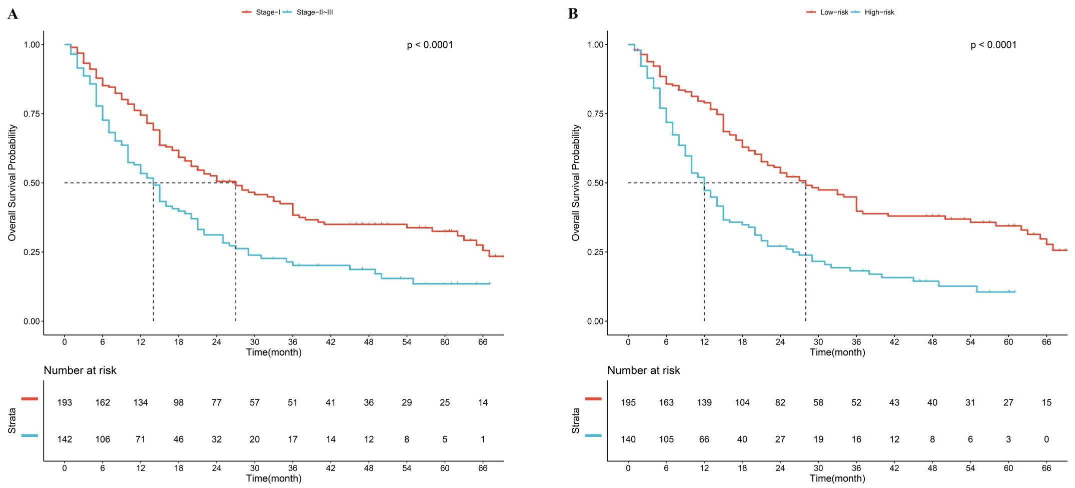

Supplement: Supplementary file 1 — Supplementary figure. [file jcav11p4115s1.pdf]
